# Construction of a High-Density Linkage Map and QTL Fine Mapping for Growth- and Sex-Related Traits in Channel Catfish (*Ictalurus punctatus*)

**DOI:** 10.3389/fgene.2019.00251

**Published:** 2019-03-26

**Authors:** Shiyong Zhang, Xinhui Zhang, Xiaohui Chen, Tengfei Xu, Minghua Wang, Qin Qin, Liqiang Zhong, Hucheng Jiang, Xiaohua Zhu, Hongyan Liu, Junjie Shao, Zhifei Zhu, Qiong Shi, Wenji Bian, Xinxin You

**Affiliations:** ^1^BGI Education Center, University of Chinese Academy of Sciences, Shenzhen, China; ^2^National Genetic Breeding Center of Channel Catfish, Freshwater Fisheries Research Institute of Jiangsu Province, Nanjing, China; ^3^The Jiangsu Provincial Platform for Conservation and Utilization of Agricultural Germplasm, Nanjing, China; ^4^Shenzhen Key Lab of Marine Genomics, Guangdong Provincial Key Lab of Molecular Breeding in Marine Economic Animals, BGI Academy of Marine Sciences, BGI Marine, Beijing Genomics Institute, Shenzhen, China; ^5^BGI-Zhenjiang Institute of Hydrobiology, Zhenjiang, China

**Keywords:** channel catfish, linkage map, quantitative trait locus (QTL), growth-related genes, sex-related marker

## Abstract

A high-density genetic linkage map is of particular importance in the fine mapping for important economic traits and whole genome assembly in aquaculture species. The channel catfish (*Ictalurus punctatus*), a species native to North America, is one of the most important commercial freshwater fish in the world. Outside of the United States, China has become the major producer and consumer of channel catfish after experiencing rapid development in the past three decades. In this study, based on restriction site associated DNA sequencing (RAD-seq), a high-density genetic linkage map of channel catfish was constructed by using single nucleotide polymorphisms (SNPs) in a F_1_ family composed of 156 offspring and their two parental individuals. A total of 4,768 SNPs were assigned to 29 linkage groups (LGs), and the length of the linkage map reached 2,480.25 centiMorgans (cM) with an average distance of 0.55 cM between loci. Based on this genetic linkage map, 223 genomic scaffolds were anchored to the 29 LGs of channel catfish, and a total length of 704.66 Mb was assembled. Quantitative trait locus (QTL) mapping and genome-wide association analysis identified 10 QTLs of sex-related and six QTLs of growth-related traits at LG17 and LG28, respectively. Candidate genes associated with sex dimorphism, including *spata2, spata5, sf3, zbtb38*, and *fox*, were identified within QTL intervals on the LG17. A sex-linked marker with simple sequence repeats (SSR) in *zbtb38* gene of the LG17 was validated for practical verification of sex in the channel catfish. Thus, the LG17 was considered as a sex-related LG. Potential growth-related genes were also identified, including important regulators such as *megf9, npffr1*, and *gas1*. In a word, we constructed the high-density genetic linkage map and developed the sex-linked marker in channel catfish, which are important genetic resources for future marker-assisted selection (MAS) of this economically important teleost.

## Introduction

Genetic-map construction is a critically important tool for further genomic studies, as well as for genetic breeding of economically important aquatic species. It has been employed for genome assembly (Jiao et al., 2014), comparative genome analysis (Xiao et al., 2015; Zhu et al., 2015; Peng et al., 2016), and quantitative trait locus (QTL) identification for important economic traits (Liu F. et al., 2013; Zhang et al., 2018). In order to construct a genetic linkage map, it is necessary to develop a large number of molecular markers on examined families. Most of the early genetic linkage map constructions used amplified fragment length polymorphism (AFLP) and simple sequence repeats (SSR), but these maps had few molecular markers with low density (Zhang et al., 2011), which limited identification of QTL and related researches.

With the rapid development of next-generation sequencing (NGS), an increasing number of methodologies have been applied for cost-effective development and genotyping of thousands of single nucleotide polymorphisms (SNPs) in non-model animals, such as genome resequencing (Lijavetzky et al., 2007), transcriptome sequencing (Triwitayakorn et al., 2011; Xiao et al., 2015), genotyping-by-sequencing (GBS) (Poland et al., 2012), restriction site associated DNA sequencing (RAD-seq), and specific-locus amplified fragment (SLAF) sequencing (Sun X. et al., 2013). At present, the RAD-seq technology is a popular tool for establishment of high-density genetic linkage maps in many aquaculture species, such as Zhikong scallop (*Chlamys farreri*) (Jiao et al., 2014), mandarin fish (*Siniperca chuatsi*) (Lijavetzky et al., 2007), tilapia (*Oreochromis niloticus* L.) (Palaiokostas et al., 2013b), Asian seabass (*Lates calcarifer*) (Wang L. et al., 2015), and Chinese mitten crab (*Eriocheir sinensis*) (Cui et al., 2015).

High-quality genetic linkage maps can locate QTLs on corresponding genomes and facilitate marker-assisted selection (MAS) and breeding in many economically important aquaculture species. Sex is one of the most basic characteristics of organisms. Many species of teleost fish have sexually dimorphic growth patterns (Tong and Sun, 2015) with significant growth differences between male and female individuals, such as yellow catfish (*Pelteobagrus fulvidraco*) (Liu H. et al., 2013), Japanese flounder (*Paralichthys olivaceus*) (Van Ooijen, 2011), half-smooth tongue sole (*Cynoglossus semilaevis*) (Song et al., 2012), and Atlantic halibut (*Hippoglossus hippoglossus*) (Palaiokostas et al., 2013a). Growth is also one of the most important economic traits for aquaculture fish species, was reported to be controlled by multi-gene and environmental effects (Feng et al., 2018) with extensive studies in many aquaculture fish species, such as rainbow trout (Sundin et al., 2005), salmon (*Salmo salar*) (Norman et al., 2012), and common carp (*Cyprinus carpio*) (Peng et al., 2016). In addition to QTL for growth and sex, QTL for stress responses, disease resistance, and cold tolerance have been mapped in other fish species (Ozaki et al., 2001; Cnaani et al., 2003; Fuji et al., 2006; Tripathi et al., 2009).

Traditional strategies for genetic improvement of growth-related traits have mainly relied on phenotypic data, which increased time and cost for breeding. However, MAS using marker-linked QTLs has accelerated genetic improvement with high accuracy of selection. Since genetic linkage map and QTLs allow to identify molecular markers or candidate genes associated with traits (Mackay et al., 2009; Feng et al., 2018), they have become important MAS breeding tools in recent years.

As an important aquaculture species, channel catfish (*Ictalurus punctatus*) has been popular in the worldwide. Especially in the native United States, it accounts for more than 60% of the US annual aquaculture production (Liu, 2011). Since its introduction to China in 1984, it has been promoted to many provinces in China, with an annual production of more than 200,000 tons. In the past decades, several linkage map and QTL studies (Waldbieser et al., 2001; Liu et al., 2003; Kucuktas et al., 2009; Li et al., 2015; Zeng et al., 2017) have been carried out to facilitate channel catfish genetic improvement and breeding programs. Previous reports confirmed that males of channel catfish grow generally faster than females under same culturing conditions (Beaver et al., 1966). Therefore, all-male monosex channel catfish has important economic values for development in aquaculture. A sex-linked marker for American strains of channel catfish was identified and the electrophoretic bands of PCR products were characterized (Ninwichian et al., 2012); however, the DNA variations of this marker should be further illustrated for validation in China strains. Meanwhile, it is necessary to establish a MAS breeding program for the channel catfish beforehand to improve the targeted economical traits. In this study, RAD-seq was employed to construct a high-density genetic linkage map, which was useful for subsequent construction of chromosome maps and identification of candidate sex-related and growth-related genes. Our present work confirms that a high-density genetic linkage map can provide a powerful tool for QTL fine mapping and genome-wide association study of economical traits.

## Materials and Methods

### Sample Collection and DNA Isolation

A F_1_ full-sib family of channel catfish was generated at National Genetics Breeding Center of Channel Catfish in Nanjing, Jiangsu Province, China, during June of 2015. Fertilized eggs were hatched with slow-flowing water (23–27°C) in separate tanks. After approximately 1 week, a random collection of approximately 5,000 larvae was stocked in separate larvae-culture tanks (3.0 m × 1.0 m × 0.5 m). Zooplankton was fed at the first 10 days, and then with formula feed. After 20 days, a random collection of approximately 1,000 larvae was transferred to an outdoor pond (about 667 square meters) for further culturing. Until December 2016, these offspring individuals at the age of 18 months were measured for growth-related traits including body height (BH), body length (BL), body weight (BW), and body width (WD), and the genders of these F1 individuals were also identified at the same time.

After investigating the relationships among the growth-related traits with R3.3.1 software (Baayen, 2008) to calculate Pearson correlation coefficients, we randomly picked up 156 healthy individuals for sample collection. Fin clips of their parents and muscle tissues of these offspring were collected in absolute ethanol, and then stored in a -20°C freezer before use. Genomic DNA was extracted using the established phenol-chloroform protocol (Taggart et al., 1992). DNA quality was evaluated via the Qubit Fluorometer (Invitrogen, United States) and electrophoresis on a 0.6% agarose gel. All experiments were performed in accordance with the Regulations of the Animal Ethics Committee and were approved by the Institutional Review Board on Bioethics and Biosafety of Freshwater Fisheries Research Institute of Jiangsu Province (No. FT 18134).

### Construction and Sequencing of RAD Libraries

DNAs of the total 158 individuals (156 offspring and two parents) were used to construct RAD libraries, which was prepared via a previously published protocol (Baird et al., 2008). In brief, each enzyme reaction system (30 μL), containing 1 μg of genomic DNA and 15 U of *EcoRI* (15 U/μL, with the restriction site of 5′GˆAATTC 3′) (Thermo Scientific, Waltham, MA, United States), was incubated at 65°C for 10 min. Barcode adapters with a sample-specific nucleotide code were designed, following the standard Illumina adapters design flow. Unique barcode adapter (10 μmol) for each DNA sample was added to individual reaction system. Twelve DNA samples were pooled per tube. In order to obtain more sequencing data for two parents, they were pooled in triplicates. Therefore, there were 162 DNA samples used for pooling. Fourteen pools were collected and the fragments at the size range of 300–500 bp were chosen. Fourteen libraries were independently sequenced using the 150-bp pair-end sequencing method on an Illumina HiSeqX-ten platform (Illumina, San Diego, CA, United States).

### Filtering of Raw Data and Splitting of Barcode Reads

After removal of sequencing adapters, low-quality reads (more than five positions with quality value less than 20, or more than 3% of unknown nucleotides) were filtered using the SOAPnuke software (Chen et al., 2018). The remained high-quality reads were used for subsequent analysis. Clean reads from the same library were separated via a Perl script on the basis of their individual barcodes. Meanwhile, those reads with wrong barcodes (not matching to the expected) were also discarded.

### SNP Discovery and Genotyping

Genome-wide SNPs were identified using a stringent SNP discovery filtering method within the software SOAPsnp (Li et al., 2009a). In order to obtain specific SNPs of the parents and the offspring, we employed SOAP2.22 (Li et al., 2009b) to align these high-quality paired-end RAD reads to the channel catfish reference genome, which was published previously (Chen et al., 2016). Based on our SOAP alignment results, we used SOAPsnp v1.05 to call SNPs. For quality control, we applied many criteria to filter SNPs, including (1) nucleotide quality more than 20; (2) depth between 3 and 300; (3) removal of reads that mapped to multiple sites; and (4) at least one heterozygote from parents. These identified SNP loci were finally separated into three segregation patterns, type lm × ll or nn × np (1:1) or hk × hk (1:2:1).

### Construction of the High-Density Genetic Map

Linkage groups (LGs) were assigned using JoinMap4.1 software (Ooijen, 2006) under the CP algorithm, and Lep-Map (Rastas et al., 2013) was used to realize the genetic map construction. First, the Lep-Map filtering module was used to filter out markers via comparison of the offspring genotype distribution and the expected Mendelian proportions (segregation distortion test). The default value of the data tolerance (*P*-value = 0.01) was used to discard highly segregated markers (χ^2^ test, *P* < 0.01). Subsequently, the separate LGomosomes module was used to assign markers into LGs and then execute with logarithm of odds (LOD) scores for recombination fraction. A range of LOD scores from 5 to 15 incrementing by 1 were tested for linkage grouping. The final LOD score (15) was selected based on whether the number of LGs matched the number of chromosomes of channel catfish and the assembled chromosomes showed the 1:1 synteny relationship with the Liu et al. (2016) map. Finally, the Order Markers module was used to orientate markers within each LG, and the Kosambi mapping function was used to convert the recombination frequencies into map distances in centiMorgans (cM) (Kosambi, 2016).

### QTL Mapping for Growth- and Sex-Related Traits

We employed MapQTL6.0 (Van Ooijen, 2011) to perform QTL analyses following the method of multiple QTL model (MQM). Regression algorithm was used for mapping quantitative trait loci in line crosses. The threshold for QTL significance was determined using a genome-wide permutation test with 200 iterations, and cofactors for MQM analyses were automatically selected with a *p*-value of 0.02. Significant LOD thresholds were calculated by permutation test of α < 0.05 and *n* = 1,000 for significant linkages. The software also calculated phenotypic variation that resulted from growth- and sex-related QTLs. These markers were mapped on the channel catfish genome (Chen et al., 2016) and upstream and downstream coding genes were identified and subsequently searched against the database of non-redundant protein sequences (Nr, cut-off value of 1e-10) at the National Center for Biotechnology Information (NCBI) using BLASTx to predict functions of these genes.

### Genome Scaffold Assembly, Synteny Analysis, and Identification of Potential Sex-Related and Growth-Related Genes

Single nucleotide polymorphisms in the genetic linkage map were used for assembling of pseudo-chromosomes. To increase the accuracy of pseudo-chromosomes assembly, we chose at least two SNPs in each scaffold using custom Perl scripts. Based on genetic distances between SNP markers, we determined the position and orientation of each scaffold and anchored these scaffolds to construct pseudo-chromosomes. To perform the genome synteny analysis, genome sequences of zebrafish (*Danio rerio*) and channel catfish (Liu et al., 2016) were downloaded from the NCBI. Genome-wide alignments were performed using lastz software (Kurtz et al., 2004), and the best homology segments were selected using perl scripts. The final genomic synteny was visualized using the Circos software (Krzywinski et al., 2009).

### Identification, Verification, and Localization of a Sex-Specific Marker

According to a previous report (Ninwichian et al., 2012), we obtained a 192-bp sex-related sequence of channel catfish by Sanger sequence. The specific primers, SexF (5′-TGAATGTGAGACTAACAGGAG-3′) and SexR (5′-ACATCGCTTTGAGAAGCTGCT-3′), were designed based on flanking sequences of this sex-linked marker using Primer3 (Koressaar and Remm, 2007) software. The forward primer was labeled with a fluorescent dye 5′6-FAM by Sangon Biotech Co. Ltd. (Shanghai, China). Subsequently, the designed specific primers were used for PCR amplification in 43 male and 53 female channel catfish individuals from two breeding populations. Each PCR reaction was done in a 20-μL volume containing 1 μL of 30–50 ng of genomic DNA, 1 μL of forward primer and reverse primer (1.0 pmol/L), 10 μL 2 × Taq PCR MasterMix [0.1 U Taq polymerase μL–1, 5.0 × 10^-4^ mol/L dNTP each, 2.0 × 10^-2^ mol/L Tris-HCl (pH 8.3), 0.1 mol/L KCl, 3.0 × 10^-3^ mol/L MgCl_2_; Vazyme, Nanjing, China], and sufficient ddH_2_O. Touchdown PCR was initiated at 94°C for 30 s. The annealing temperature of these reactions decreased from 60°C to a touchdown 50°C at the cooling rate of 1°C in every cycle; followed 94°C for 30 s, 55°C for 30 s, 72°C for 30 s, 20 cycles; and final extension steps at 72°C for 10 min. For fragment length analysis, PCR products were genotyped on a ABI PRISM 3730XL DNA Sequencer (Applied Biosystems, Foster City, CA, United States) with GS500 marker as an internal size standard. The allelic sizes were determined using GeneMarker version 1.5 (SoftGenetics LLC, State College, PA, United States).

## Results

### Characteristics of the Growth- and Sex-Related Traits

Individuals in the mapping family had an average BH of 6.69 ± 0.78 cm, an average BL of 30.05 ± 2.93 cm, an average BW of 601.76 ± 187.53 g, and an average WD of 5.18 ± 0.59 cm. The growth-related traits showed strong correlation with each other (*r* = 0.8292-0.9312, *P*-value* <* 0.001 for all). The highest correlation value 0.9312 was observed between BW and BL (Table 1). In the mapping population, 74 and 82 individuals were identified as male and females, respectively, with a sex ratio of 1:1.11. Statistics on male and female growth data demonstrated that male individuals have larger BL, BH, body wide, and BW than female individuals (Table 2), indicating that males grow faster than females under the same culturing condition.

**Table 1:** Pearson correlation coefficients for all pairwise combinations of the four examined traits.

Traits	BW	BL	BH	WD
BW	1	0.9312	0.8747	0.8847
BL	0.9312	1	0.8292	0.8318
BH	0.8747	0.8292	1	0.8612
WD	0.8847	0.8318	0.8612	1


**Table 2:** Significant growth difference between female and male channel catfish.

Item	Male (mean)	Female (mean)	*P*-value (*t*-test)
BW (g)	643 ± 205.8^∗∗^	563 ± 161.4	0.007
BL (cm)	32.59 ± 3.22^∗^	31.56 ± 2.57	0.027
BH (cm)	6.83 ± 0.83^∗^	6.57 ± 0.72	0.038
WD (cm)	5.29 ± 0.59^∗^	5.08 ± 0.56	0.022


*^∗^Significant difference; ^∗∗^very significantly different.*

### Summary of the RAD Libraries

A total of 14 RAD-seq libraries from two parents and their 156 offspring individuals were sequenced on an Illumina HiSeqX-ten platform to generated about 1.59 billion of 150-bp pair-end reads. The DNA sequencing raw data have been deposited for public availability in CNSA (CNGB Nucleotide Sequence Archive)^1^ with the project no. CNP0000229. After subsequent filtering of low-quality reads, 1.42 billion of clean reads were remained. The number of clean reads per offspring individual was averaged to 8.66 million. Meanwhile, both the female and male parental data contained 28.76 and 25.64 million of clean reads, respectively.

### SNP Calling and Construction of the High-Resolution Genetic Linkage Map

A total of 1,367,192 SNPs in all individuals were identified using SOAPSnp, in which 10,661 SNPs passed through the filtering criteria (see more details in section “Materials and Methods”). These SNPs were classified into three categories: paternal heterozygous (lm × ll, 5,617 SNPs), maternal heterozygous (nn × np, 4,915 SNPs), and heterozygous in both (hk × hk, 129 SNPs). Among them, 4,768 SNPs were consistent with Mendelian segregation pattern, and then they were used for subsequent linkage map construction using a pseudo-testcross strategy (Shao et al., 2015).

**FIGURE 1:**
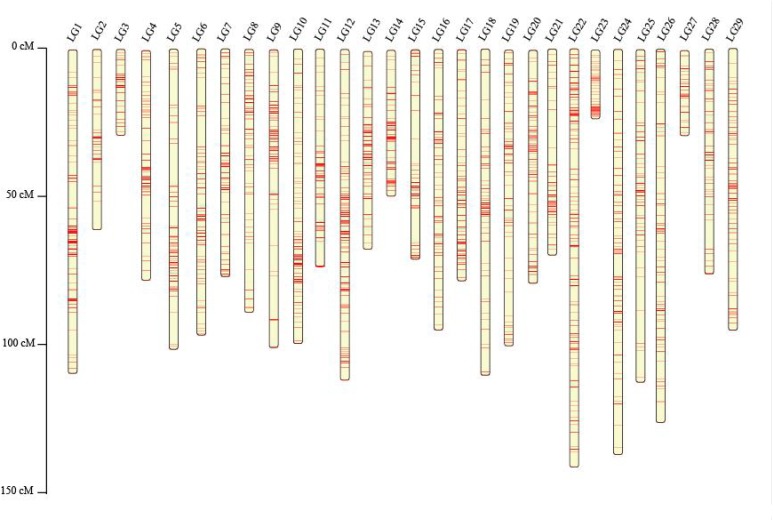
The genetic linkage map of Chinese channel catfish constructed based on SNP markers.

These SNPs were finally grouped into 29 LGs (Table 3 and Figure 1), which is consistent with the reported haploid chromosome number of the channel catfish (Liu et al., 2016). The genetic linkage map spanned 2,480.25 cM with an average SNP interval of 0.55 cM. The genetic length of each LG ranged from 28.71 (LG27) to 141.1 cM (LG22), with an average SNP distance of 0.21–1.01 cM (Table 3 and Figure 1).

**Table 3:** Characteristics of genetic linkage map and anchoring scaffolds of channel catfish.

LG_ID	Length of anchored scaffolds (Mb)	No. of anchored genes	No. of anchored scaffolds	No. of SNPs	Distance (cM)	Average inter-loci distance (cM)
LG1	32.97	939	22	308	109.18	0.35
LG2	11.3	300	3	67	60.85	0.91
LG3	25.4	643	6	138	29.01	0.21
LG4	16.9	501	5	186	77.46	0.42
LG5	22.99	647	7	153	101.21	0.66
LG6	22.73	595	11	183	96.57	0.52
LG7	25.49	714	11	144	76.81	0.53
LG8	24.2	661	4	156	88.71	0.56
LG9	31.5	783	12	175	100.47	0.57
LG10	33.31	743	7	205	99.39	0.48
LG11	18.72	408	5	143	73.32	0.51
LG12	22.8	622	10	419	111.54	0.27
LG13	40.9	961	10	200	66.76	0.33
LG14	36.44	905	9	171	49.21	0.28
LG15	18.73	411	11	143	70.69	0.49
LG16	7.23	215	1	166	94.6	0.56
LG17	30.04	655	10	181	78	0.43
LG18	32.68	864	9	155	110.03	0.7
LG19	28.52	837	4	120	99.71	0.83
LG20	22.94	649	12	141	78.59	0.55
LG21	14.47	438	2	111	69.52	0.62
LG22	32.78	618	6	318	141.1	0.44
LG23	16.91	402	3	63	23.23	0.36
LG24	33.41	790	7	152	136.71	0.89
LG25	17.44	475	8	136	112.19	0.82
LG26	23.50	614	5	125	125.9	1.01
LG27	8.43	189	5	62	28.71	0.46
LG28	22.67	788	8	116	75.76	0.65
LG29	29.17	794	10	131	95.02	0.72
Total	704.66	18,161	223	4,768	2,480.25	-
Average	24.29	626	7	164	85.52	0.55


*The anchored genes and scaffolds are from the genome assembly (Chen et al., 2016).*

### Fine QTL Mapping for Growth- and Sex-Related Traits

**FIGURE 2:**
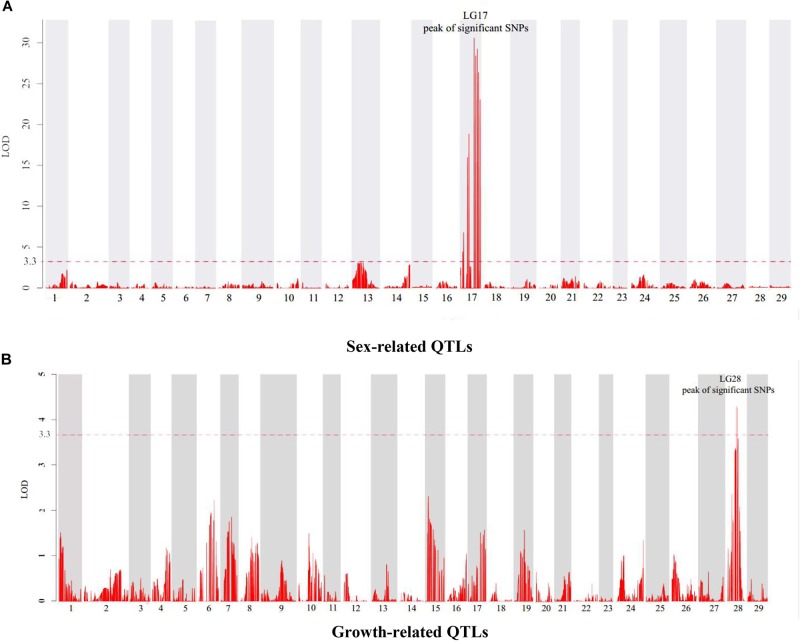
Genetic location of QTLs for sex **(A)** and growth **(B)** in the genetic linkage map of channel catfish. The red dashed line represents a linkage group-wise logarithm of odds (LOD) significance threshold of 3.3.

In this study, four growth-related traits including BW, BL, BH, and WD were measured. Six QTLs associated with growth-related traits were identified on the LG28, which were detected between the narrow span of 32.53–45.29 cM (Table 4). Among these QTLs, the highest LOD value of 4.29 (Figure 2A) was located at 37.46 cM near the marker Scaffold53-3609071, which accounts for 11.8% of the phenotypic variation. No major locus explaining > 20% of the total variation was detected among these growth-related QTLs.

**Table 4:** Characteristics of the growth- and sex-related QTLs in the channel catfish (LOD > 3.3).

Trait	QTL	LG	Nearest marker	LOD	CI (cM)	% Expl
Sex	qSEX_1	17	Scaffold134-267434	4.50	9.35–10.18	12.3
	qSEX_2	17	Scaffold60-2211815	23.62	39.11–40.11	49.8
	qSEX_3	17	Scaffold60-3032257	16.19	27.66–28.26	37.6
	qSEX_4	17	Scaffold60-2727790	19.1	33.19–33.69	42.7
	qSEX_5	17	Scaffold55-2157602	31.01	53.39	59.5
	qSEX_6	17	Scaffold60-1735567	28.8	59.77	56.8
	qSEX_7	17	Scaffold11-9389916	29.63	65.54	57.8
	qSEX_8	17	Scaffold11-8795560	26.73	70.31	54.1
	qSEX_9	17	Scaffold11-3807312	23.36	76.38–76.92	49.4
	qSEX_10	17	Scaffold11-2572525	23.14	74.38–75.38	49.1
Growth	qGW_1	28	Scaffold53-3650014	3.33	32.53–32.74	9.3
	qGW_2	28	Scaffold39-5862985	3.30	34.76–34.86	9.1
	qGW_3	28	Scaffold52-3364243	3.32	35.53	9.2
	qGW_4	28	Scaffold53-3609071	4.29	37.46	11.8
	qGW_5	28	Scaffold39-2407434	4.27	42.30–43.06	11.7
	qGW_6	28	Scaffold39-2138154	3.58	45.20–45.29	9.9


*GW, growth; Expl, percentage of explained phenotypic variation.*

Meanwhile, 10 significant QTLs for sex determination were detected on the LG17 of the channel catfish using permutation tests (*P* < 0.02, LOD > 3.3). Among these QTLs, the highest LOD value of 31.01 (Figure 2B) was located at 53.39 cM near the marker Scaffold55-2157602, which contributed to 59.5% of the phenotypic variation.

### Chromosomal Assembly and Comparative Genome Analysis

Twenty-nine pseudo-chromosomes (Chr) of channel catfish with a total length of 704.66 Mb were assembled, which comprised 83.39% of the assembled scaffold sequences (Chen et al., 2016) and 18,161 genes (a total number of 21,556 genes). The average pseudo-chromosome length was 24.29 Mb with seven scaffolds. The smallest pseudo-chromosome was chr16 with 7.23 Mb containing one scaffold, and the largest pseudo-chromosome was chr13 with 40.9 Mb containing 10 scaffolds.

**FIGURE 3:**
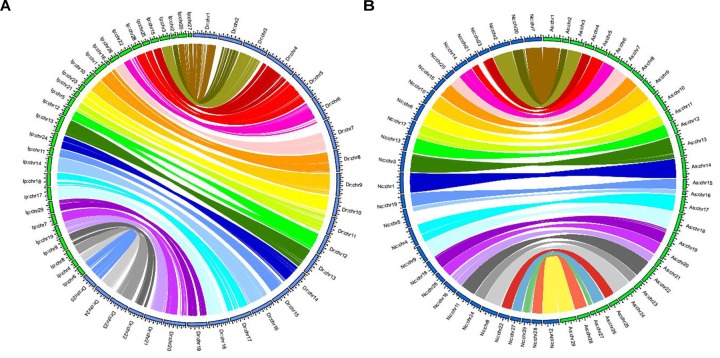
**(A)** Circos diagram representing syntenic relationships between channel catfish (Ip: *Ictalurus punctatus*, assembled by Chen et al., 2016) (green) and zebrafish (Dr: *Danio rerio*) (blue). **(B)** Circos diagram representing syntenic relationships between two assembled channel catfish chromosomes (green, assembled by us; blue, from Liu et al., 2016).

**FIGURE 4:**
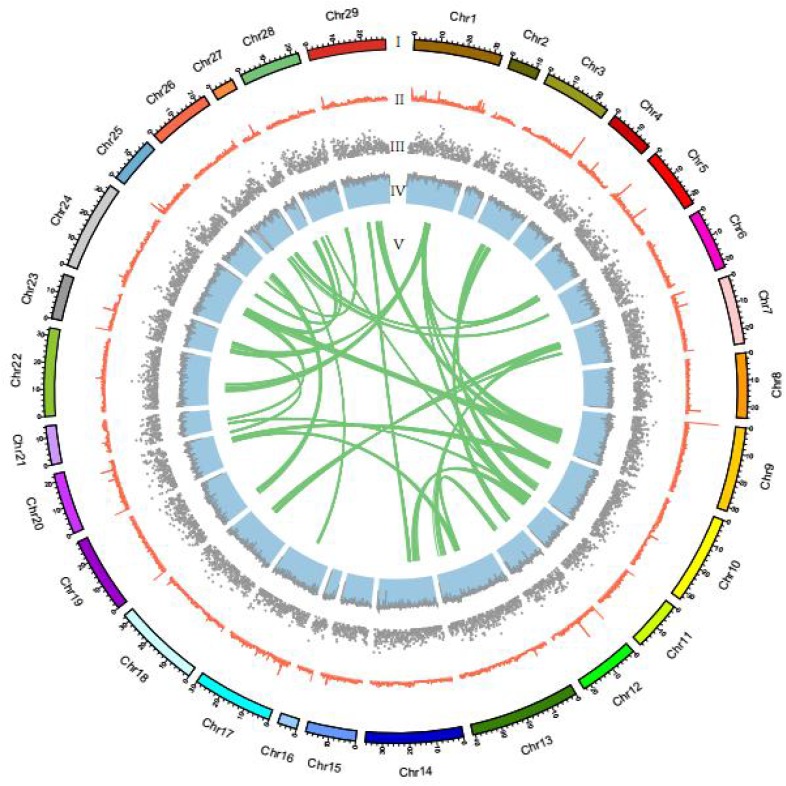
Circos atlas representation of the pseudo-chromosome information. (I) The length of each pseudo-chromosome. (II) Density of SNP distribution in each 100-kb genomic interval. (III) Density of gene distribution in each 100-kb genomic interval. (IV) GC content of 100-kb genomic intervals. (V) Schematic presentation of major interchromosomal relationships in the channel catfish genome.

There were 16,197 synteny blocks between the assembled genomes of channel catfish and zebrafish, and 15 of the 29 LGs of channel catfish had relatively conserved collinear blocks on zebrafish chromosomes (Figure 3A). The total number of synteny blocks between our channel catfish assembly (Chen et al., 2016) and the assembly published by Liu et al. (2016) was 55,525. All chromosomes showing the 1:1 synteny relationship (Figure 3B). Figure 4 summarizes the distribution of SNPs, genes, GC content on 100-kb genomic intervals, and interchromosomal relationships of our assembled channel catfish pseudo-chromosomes.

### Potential Candidate Genes for Sex Dimorphism and Growth-Related Traits

**FIGURE 5:**
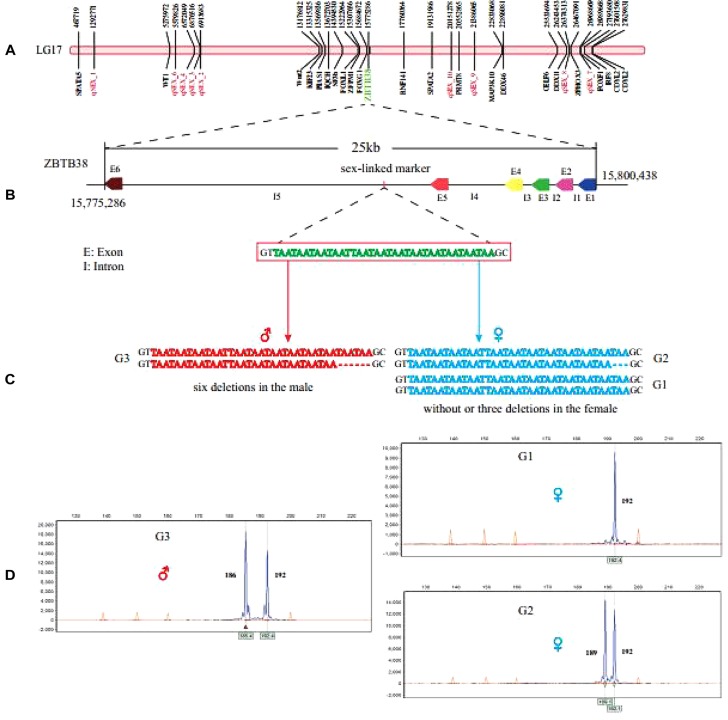
Sex-related QTLs and gene distribution on the LG17. **(A)** Highlighted red are QTLs linked to sex. **(B)** The location of sex-linked marker on the *zbtb38* was determined. **(C)** Sex-linked markers for genotypes in both male and female individuals, heterozygous (six nucleotides deletion) in males and homozygous or heterozygous (three nucleotides deletion) in females, were validated. **(D)** The sex-linked marker was verified using genotyping in other channel catfish groups.

To further identify potential genes underlying sex dimorphisms, we used BLASTX to search gene sequences on the LG17 from the QTL regions against the NCBI Nr database. Finally, 23 sex-related genes were predicted, and they were previously reported to be involved in spermatogenesis, gonad sex determination, and testicular determination (Table 5). These genes included spermatogenesis-associated protein 2 (*spata2*), *spata5*, splicing factor 3 (*sf3*), and forkhead box (*fox*). Five sex-related genes including Wilms tumor protein 1-interacting protein (*wt1*), *spata2*, probable ATP-dependent DNA helicase DDX11 (*ddx11*), zinc finger homeobox protein 3-like isoform X1 (*zfhox3*), and forkhead box protein F1 (*foxf1*) were located near four sex-related QTLs (qSEX_6, qSEX_7, qSEX_8, and qSEX_10) (Figure 5A).

**Table 5:** Annotation of growth- and sex-related candidate genes in the genome of channel catfish.

Gene ID	LG	Abbreviations	Gene name in nr database	Reference
CF_GLEAN_10001855	LG17	*spata5*	Spermatogenesis-associated protein 5	Sun F. et al., 2013
CF_GLEAN_10004009	LG17	*foxg1*	Forkhead box protein G1-like	Hu et al., 2017
CF_GLEAN_10004013	LG17	*zbtb38*	Zinc finger and BTB domain-containing protein 38	Couderc et al., 2002
CF_GLEAN_10009162	LG17	*kif23*	Kinesin-like protein KIF23 isoform X1	Sun F. et al., 2013
CF_GLEAN_10009169	LG17	*iqch*	IQ domain-containing protein H isoform X1	Sun F. et al., 2013
CF_GLEAN_10009212	LG17	*foxl1*	Forkhead box protein L1	Hu et al., 2017
CF_GLEAN_10009215	LG17	*zfpm1*	Zinc finger protein ZFPM1	Mardon and Page, 1989
CF_GLEAN_10016328	LG17	*cdyl2*	Chromodomain Y-like protein 2	Lahn et al., 2001
CF_GLEAN_10016329	LG17	*cdyl2*	Chromodomain Y-like protein 2	Lahn et al., 2001
CF_GLEAN_10016347	LG17	*irf8*	Interferon regulatory factor 8 isoform X1	Martinez et al., 2014
CF_GLEAN_10016349	LG17	*foxf1*	Forkhead box protein F1	Hu et al., 2017
CF_GLEAN_10016370	LG17	*zfhox3*	Zinc finger homeobox protein 3-like isoform X1	Mardon and Page, 1989
CF_GLEAN_10016375	LG17	*ddx11*	Probable ATP-dependent DNA helicase DDX11	Sun F. et al., 2013
CF_GLEAN_10016428	LG17	*ddx46*	Probable ATP-dependent RNA helicase DDX46	Sun F. et al., 2013
CF_GLEAN_10016485	LG17	*prmt8*	Protein arginine N-methyltransferase 8-B-like	Sun F. et al., 2013
CF_GLEAN_10016501	LG17	*spata2*	Spermatogenesis-associated protein 2-like protein	Chen et al., 2015
CF_GLEAN_10007358	LG17	*wt1*	Wilms tumor protein 1-interacting protein	Lin et al., 2017
CF_GLEAN_10009192	LG17	*sf3b*	Splicing factor 3B subunit 3	Lin et al., 2017
CF_GLEAN_10009153	LG17	*wnt2*	Protein Wnt-2	Luo et al., 2015
CF_GLEAN_10016543	LG17	*rnf141*	Ring finger protein 141	Sun F. et al., 2013
CF_GLEAN_10016386	LG17	*celf6*	CUGBP Elav-like family member 6	Blech-Hermoni and Ladd, 2015
CF_GLEAN_10016430	LG17	*map3k10*	Mitogen-activated protein kinase kinase kinase 10	Bogani et al., 2009
CF_GLEAN_10009166	LG17	*pias1*	E3 SUMO-protein ligase PIAS1	Oh et al., 2007
CF_GLEAN_10009228	LG28	*gas1*	Growth arrest-specific protein 1	Del Sal et al., 1992
CF_GLEAN_10009299	LG28	*megf9*	Multiple epidermal growth factor-like domains protein 9	Thakker et al., 2018
CF_GLEAN_10009247	LG28	*npffr1*	Neuropeptide FF receptor 1	Peng et al., 2016


We screened our reference genome and collected protein-coding genes from the growth-related QTL regions. Three growth-related genes, including multiple epidermal growth factor-like domains protein 9 (*megf9*), neuropeptide FF receptor 1 (*npffr1*), and growth arrest-specific protein 1 (*gas1*), were found in two (qGW_1 and qGW_3) of the six growth-related QTL regionso (Table 5). Among these genes, *npffr1* is receptor of neuropeptide FF and RFamide-related peptide (*rfrp*), which are involved in control of feeding behavior both in certain invertebrates and in vertebrates (Dockray, 2004; Bechtold and Luckman, 2007).

These candidate genes localized on the LG17 and LG28 may involve in the genetic control of sex- and growth-related traits. Detailed functions are worthy of further investigation for genetic breeding of channel catfish.

### A Sex-Linked Marker Was Verified in the Channel Catfish

According to previously reported molecular marker (Ninwichian et al., 2012), we obtained a 192-bp fragment by Sanger sequencing. Subsequently, we used the clean reads of the samples in this study to perform multiple sequence alignments with this special sex-linked sequence. We observed that the sex-linked locus consists of three types of allele, including no deletion (allele G1), 3-bp deletion [one (TAA) repeat; allele G2], and 6-bp deletion [two (TAA) repeats; allele G3] in the SSR marker, respectively (Figure 5C,D).

The 6-bp deletion allele presented in all male individuals but not in any of the females (Figure 5C,D), thus it could definitely distinguish male and female individuals. Chromosome location of this sex-linked SSR marker clearly marked it on the LG17 of channel catfish, within the non-coding region of zinc finger and BTB domain-containing protein 38 (*zbtb38*), which is approximately 25 kb in length and consists of six exons and five introns (Figure 5B). Furthermore, to verify the sex-linked SSR marker, we designed fluorescent primers to perform PCR amplification in 43 male and 53 female catfish individuals from two other breeding populations. The PCR results matched phenotypes with a 100% overall accuracy.

## Discussion

A genetic linkage map can provide important genomic information and allow for exploration of QTL, which can be used to maximize the selection of target traits in breeding animals. Availability of a large number of genetic markers is essential for constructing a good genetic linkage map and for QTL mapping of available genetic traits. High-density linkage maps and growth/sex-related QTLs were analyzed using RAD sequencing in several aquatic animals, such as genetic linkage map construction in orange-spotted grouper (*Epinephelus coioides*) (4,608 SNPs) (You et al., 2013), genetic map and sex-/growth-related QTL in turbot (*Scophthalmus maximus*) (6,647 SNPs) (Wang W. et al., 2015), blunt snout bream (*Megalobrama amblycephala*) (14,648 SNPs) (Wan et al., 2017), mandarin fish (*S. chuatsi*) (3,283 SNPs) (Sun et al., 2017), common carp (*C.c. haematopterus*) (7,820 SNPs and 295 SSRs) (Feng et al., 2018), and pompano (*Trachinotus blochii*) (12,358 SNPs) (Zhang et al., 2018).

In the present study for channel catfish, we employed the RAD sequencing technology to construct a high-density genetic linkage map with 4,768 SNPs, reaching the total length to 2,480.25 cM with an average SNP distance of 0.55 cM. Using the markers to target specific scaffolds from previous study (Chen et al., 2016), we anchored a total number of 223 scaffolds in the channel catfish genome assembly to 29 LGs. Approximately 704.66 Mb (83.39%) of the assembled genome sequences were assigned to the 29 LGs with identification of 18,161 genes (84.25%). Interestingly, in previous work (Chen et al., 2016), a female individual collecting from a local breeding stock in China was used for genome sequencing, and the assembly genome size was 845 Mb. However, in another published study of channel catfish genome using a doubled haploid female individual from the United States (Liu et al., 2016), a 783-Mb genome was assembled. The remarkable different genome size may be generated from the different source of sequencing samples.

Many species of teleost fish have sex dimorphic growth patterns (Mei and Gui, 2015), and there are significant growth differences between males and females (Gui and Zhu, 2012; Liu F. et al., 2013).Therefore, in some species, production of monosex populations is desirable for economic values. In this study, we observed that males of the Chinese channel catfish have significant differences in BW and BH compared with females (*P*-value < 0.05, Table 2), and males exhibited much faster growth than females under the same culturing condition. This sexual dimorphism was also determined in other fish species, such as yellow catfish (*P. fulvidraco*) (Liu H. et al., 2013) and Nile tilapia (*O. niloticus*) (Lee et al., 2011). The high-density genetic linkage map generated in present study provided useful data for QTL fine mapping of important economically traits (especially growth- and sex-related) in channel catfish.

Growth and sex are the most important traits for cultured fish species. Based on established genetic linkage maps, researchers have determined many practical QTLs for sex/growth traits. For example, in mandarin fish (*S. chuatsi*) one significant QTL for sex determination was identified on LG23; genotypes of all the female fish on r1_33008 marker were heterozygous, and all males were homozygous, thus this sex-specific marker can be used to identify male and female individuals of mandarin fish; meanwhile, 11 significant QTLs for growth traits were also detected on four LGs (Sun et al., 2017). Similarly, in our present study, 11 significant QTLs associated with sex-related trait at LOD ≥ 3.3 were detected on the LG17, contributing to 12.3–59.5% of the phenotypic variation in channel catfish. This finding was confirmed in other fish species, such as Atlantic halibut (*H. hippoglossus*) (Palaiokostas et al., 2013a) and gilthead sea bream (*Sparus aurata*) (Loukovitis et al., 2011).

In contrast, sex-related QTLs of some other fish species were detected to be distributed on different LGs. In the blunt snout bream (*M. amblycephala*), three QTLs related to gonad development were detected on LG13, LG12, and LG1 (Wan et al., 2017). In the tilapia, sex-linked QTLs were detected on at least three LGs (LG1, LG3, and LG23; Lee et al., 2003; Cnaani et al., 2004; Eshel et al., 2012). These results suggest involvement of multiple chromosomes or LGs in sex relation and provide support to the polygenic sex determination in fishes. Sex determination of channel catfish probably is male-dominant (XX/XY), due to sex ratios of offspring both in interspecific hybridization (*I. punctatus* 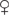 × *Ictalurus furcatus* 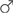) and intraspecific hybridization of channel catfish close to 1:1, and the offspring in gynogenetic families were all females (Goudie et al., 1995). Similar to other fish species, the channel catfish sex chromosomes (X and Y) is difficult to be distinguished based on current karyotype analysis technologies. Therefore, identification of sex-specific markers and QTLs is a necessary prerequisition for uncovering sex determination mechanisms and associated genes, as well as proceeding sex-transformation and sex control tests.

Sex-specific markers have been identified in more than 20 economic aquaculture species to date by the strategies of AFLP and NGS. In the present study, 23 genes related to sex development were detected on the LG17, such as *spata* (related to sperm production; Sun F. et al., 2013; Chen et al., 2015), *wt1* (early establishment of gonad; Lin et al., 2017), and *foxl1* (sex hormone regulation; Hu et al., 2017). The result is similar to tilapia, in which 51 sex-determination genes were annotated in the sex region on scaffold 101 (Eshel et al., 2012). In our current study, all sex-related QTLs are located on the same LG17 suggesting that a single chromosome may be involved in sex determination (Figure 3). To validate a previously reported sex-specific marker in channel catfish, we extracted those male-specific tags presented in all male individuals but not in any of females. Finally, we obtained one SSR marker with 6-bp deletion presented in all male *zbtb38* gene. Results from fluorescent PCR-capillary gel electrophoresis also confirmed the male-specific SSR marker. Likewise, sex-specific SSR markers have been reported in half-smooth tongue sole (*C. semilaevis*) (Chen et al., 2012) and kiwifruit (*Actinidia chinensis Planchon*) (Zhang et al., 2015), and they were used to distinguish male and female in practical breeding programs.

The good resolution and high density of our genetic linkage map provide an effective support for QTL mapping of economically traits, as well as for genome assembly. QTL fine mapping and positional cloning of candidate genes have been an efficient approach for breeding programs in aquaculture animals, especially for investigation of quantitative traits (Xiao et al., 2015; Yu et al., 2015; Peng et al., 2016). Interestingly, in our present study, six QTLs associated with growth traits (BL, BH, BW, and WD) were identified to cluster at a narrow linkage span (32.53–45.29 cM) of the LG28. We screened the reference genome (Chen et al., 2016) and identified three protein-coding genes from this growth-related QTL region, providing potential tools for molecular breeding of new variants with growth superiority.

## Conclusion

In the present study, we employed RAD sequencing to construct a high-density genetic linkage map with 4,768 SNPs for the channel catfish. Ten sex-related QTLs were detected on the LG17, on which 23 genes related to sex development were annotated, such as *spata, wt1*, and *foxl1*. Six QTLs for growth were detected on the LG28, on which three growth-related genes were identified within the QTL intervals. A previously reported sex-linked marker was confirmed on the LG17, which can effectively identify male and female individuals of channel catfish from difference genetic resources. In summary, we provide a valuable genetic resource for future molecular breeding of this economically important fish species.

## Data Availability

The datasets generated for this study can be found in CNGB Nucleotide Sequence Archive (https://db.cngb.org/cnsa/), CNP0000229.

## Author Contributions

XY, SZ, QS, WB, and XC conceived the ideas and designed the investigations. XZ, TX, and SZ analyzed the data. SZ, MW, QQ, LZ, and HJ collected and processed the samples. HL, JS, and ZZ performed the experiments. XZ, SZ, and XY wrote the manuscript. XY and QS revised the manuscript. All authors read and approved the final manuscript for publication.

## Conflict of Interest Statement

The authors declare that the research was conducted in the absence of any commercial or financial relationships that could be construed as a potential conflict of interest. The handling Editor declared a past co-authorship with one of the authors XY.

## Footnotes

1 https://db.cngb.org/cnsa/
